# Platforms and Institutions in the Post-Pandemic University: a Case Study of Social Media and the Impact Agenda

**DOI:** 10.1007/s42438-021-00269-x

**Published:** 2021-11-04

**Authors:** Mark Carrigan, Katy Jordan

**Affiliations:** 1 Institute of Education, University of Manchester, Manchester, UK; 2 Faculty of Education, University of Cambridge, Cambridge, UK

**Keywords:** Social media, Platforms, Impact, Research evaluation, Covid-19

## Abstract

In this paper, we argue that digital platforms play an important role within higher education, not least of all when Covid-19 has made remote working the norm. An increasingly rich field of theoretical and empirical work has helped us understand platforms as socio-technical infrastructures which shape the activity of their users. Their insertion into higher education raises urgent institutional questions which necessitate dispensing with the individualised mode of analysis and instrumentalised conception of technology which often accompany these topics. We outline an alternative approach through a case study of social media in the 2014 Research Excellence Framework, exploring the incorporation of platforms into research evaluation. Our findings suggest social media is invoked differently across disciplinary groupings, as well as platform metrics being cited in a naive and problematic matter. We offer a neo-institutionalist analysis which identifies a tendency towards isomorphism, with perceived ‘best practice’ being seized upon in response to uncertainty. We suggest such an approach is urgently needed given the role which digital platforms will play in building the post-Pandemic university.

## Introduction

The term ‘platform’ has become commonplace in recent years. It usually refers to digital infrastructures which enable multiple parties to interact with each other at a distance. For example, Uber coordinates the interaction of drivers and riders, eBay links buyers and sellers while Facebook facilitates friends and acquaintances to interact at a distance. It builds on what Wellman and Rainie (2012) describe as the *triple revolution* of social networks, the Internet and mobile computing. It identifies an important tendency in how innovative technologies are being put to use in products and services that are increasingly influential within the capitalist system (Srnicek 2017; Pasquale 2016). The notion of the platform helps us talk about this transformation with some degree of analytical specificity, even if there remains much work to be done. But it can also be a slippery term. As Gillespie (2010) warns us, it trades off overlapping computational, architectural, political and figurative meanings in a manner which means it can mystify the operations of platform firms. Uber claims it *merely* matches drivers with riders, denying the employment relationship it enters into and the responsibilities which flow from it. eBay claims it *merely* facilitates commercial exchanges between parties in relation to whom it has no responsibilities, denying its role in trades which would not otherwise take place. Facebook claims it *merely* connects the world, denying its role as a publisher and the responsibilities it entails. These are just a few examples of how the terminology of the platform can be deployed in self-interested ways by firms. This does not mean we should abandon it because recognising this discursive work can help us understand the operations of platform firms (Carrigan and Fatsis 2021). To ask how firms *present* what they do invites the question of how this relates (or fails to) with what they *actually* do (Gillespie 2018).

Platforms are proliferating within higher education at a dizzying rate (Carrigan 2019a; Robertson 2018). Goldenfein et al. (2019) suggest Google Scholar has enormous significance for the knowledge system in spite of the opacity which surrounds its construction of an index, extraction of bibliometrics information and development of rankings based on perceived relevance. We can see a similar opacity in platforms orientated towards researchers such as Academia.edu, ResearchGate and Mendeley (Jordan 2019). Komljenovic (2018) analyses how such platforms individualise academic work, enhance competition and restructure social relations.

Multi-sidedness is inherent to platforms, and this capacity to facilitate interaction between different parties is crucial to their operational utility (McAfee and Brynjolfsson 2017). In some cases, they facilitate a process which would have otherwise been too logistically intensive, enabling forms of exchange that simply would not have happened otherwise. In other cases, they automate a process which would otherwise have required the intervention of a human agent to facilitate the allocation of resources between parties. In this sense, we can see something like a *room booking* service as an example of a platform, even if it would seem to differ from the more familiar consumer-facing services: it mediates between those who want to use a resource and those charged with allocating a resource in order to increase the ease with which the former can make claims and the latter can administer them.

There are a wide range of university services which can be *platformised* in this way, and we still lack an overview of what appears to be a rapidly expanding market, e.g. knowledge-exchange (*In-Part*), recruitment (*Eploy*), room management (*Booker*), learning management (*Moodle*), student engagement (*Eventus*) and alumni engagement (*Ellucian*). These are just a small selection of the diverse platforms which are being launched, and mapping these offerings in a systematic way is an undertaking which is still in its infancy. In some cases, these are tailored services for universities, in other generic platforms for large organisations. The development of enterprise platforms targeted at universities is particularly significant, not least of all in terms of what Williamson (2018) documents as the building of a backend infrastructure which links them together.

These inevitably incorporate assumptions about how an organisation like a university can and should work, with real-world consequences. As McCluskey and Winter (2012: 13) observe, this involves a transfer of the ‘flexibility and power that individual faculty members, registrars and advisers once owned’ to the ‘standardised mechanism of the software’. This software of course does not run autonomously, granting a new organisational significance to those who implement, operate, modulate and update its enactment as the beating heart of the university. It supports what Nash (2019) describes as *marketising* bureaucracy, in that it standardises the experience of student consumers and routinises their interaction with the university. There are important institutional questions which need to be asked about the influence of digital platforms within the sector.

## The Common Features of Platforms and their Significance for Higher Education

There are a number of features which platforms *tend* to share, reflecting a common identity as software facilitating multi-sided coordination between distributed social actors (McAfee and Brynjolfsson 2017). Identifying these generic features can help us begin to analyse how they play out within higher education in ways which are specific to the sector:

1. **Data-generating**: a voluminous literature has developed around ‘big data’ in recent years (Mayer-Schönberger and Cukier 2013). Often defined in terms of a continually expanding list of v’s (volume, variety, velocity and veracity etc.), big data is produced as a by-product of user engagement with digital systems (Carrigan 2018). Even though ‘click trails’ long precede big data, the methodological impetus of the literature concerns the *scale* at which this data is generated, as well as the latent value understood to reside within it across a whole range of strategic arenas (Kitchin 2014). Furthermore, the volume of data accrues incredibly quickly and is often subject to analysis in real time. This is integral to the business model of platforms but it is important to recognise that, as Gitelman (2013) memorably puts it, *raw data is an oxymoron*: it is structured, filtered and selected. The velocity of this process is significant because it exceeds human analytical capacities and entails a reliance upon automation to organise the emerging data.
2. **Opacity**: while data is generated within platforms as a by-product of user activity, the proprietors of the platform enjoy a privileged relationship to that data. This entails the rapidly increased production of social data about interaction which is immediately available to one party to that interaction and not to others, described by Andrejevic (2013) as the *big data divide*. This is reflected in a dual structure in which a front-end interface orientated towards users is combined with a back-end infrastructure facilitating analysis and intervention to shape the behaviour of those users: an asymmetric relation between users whose behaviour is influenced and operators who seek to influence (Marres 2017, 2018). In this sense, users are objects of prediction rather than sources of agency, with their user behaviour treated as traces which fuel real-time analytical processes. Persuasive design is a field which has emerged around the new possibilities which these datasets afford, presenting us with a digital environment in which products and services are designed to elicit ever more involvement and engagement from us (Williams 2018).
3. **Lock-in**: platforms have a tendency to lock users into their operation in a number of ways. Firstly, their use entails resources, even if this is just the time and energy of individual users, meaning there are sunk costs over time. This investment by end users in selecting and familiarising themselves with platforms contributes to the intensification of labour within the academy (i.e. it is another professional task to be accomplished within working hours) while also providing a disincentive to leave the platform, particularly if this investment of time has been made at the level of a research project or collaborative network.^1^ This is as true of institutional operators as it is of end users, though the former have a much wider sunk costs, given the difficulty of implementing a platform, managing its uptake and developing the expertise to maintain it. Secondly, each platform has its own classificatory economy, in the sense of categories which must be learned and applied, liable to be real in their consequences purely in virtue of the fact they are operative through the platform (Marres 2017). These categories are part of the user interface and designed to encourage the user to respond in certain ways over time, including thinking and evaluating in such terms, e.g. to be enthusiastic about seeking more followers or feel gratified when a comment is retweeted.

In what follows, we treat social media as a *type* of platform. It is distinctive because of its visibility, popularity and accessibility. In contrast to many of the platforms found within the university, it has largely grown on an opt-in basis through individuals and their networks, as opposed to being sponsored by the institution itself. There are many questions which follow from these characteristics about what the university is, how work is organised within it and how its boundaries are changing (Woodcock 2018). As Bacevic (2019) analyses, they lead to a recasting of behaviour which would have once been seen as ‘private’ into a ‘public’ form in two ways. Firstly, there is a demonstrable tendency for academic speech to become more contested as academics use social media to talk in *potentially* public ways to audiences of unknown size and intention (Carrigan 2019b). Secondly, the uptake of social media inserts private firms into academic exchanges as intermediaries, with still unfolding consequences.

In the next section, we reflect on the research literature which has emerged around social media in higher education and suggest that, for all its empirical value, it is constrained by a tendency towards an *individualised* mode of analysis and an *instrumentalised* conception of technology. Unless we can dispense with these tendencies, it will remain difficult to ask the institutional questions about the future of higher education raised by the emergence of social media, as well as digital platforms more generally. In a time of Covid-19 when we are relying on these platforms to facilitate teaching, learning and administration at a distance, we urgently need to understand their implications.

## Social Media in Higher Education

In less than a decade, social media has gone from being a fringe feature of academic life to a familiar presence within the British university system.^2^ Facebook, Twitter and Instagram are established means by which universities communicate with their different stakeholders, with some universities using an even broader range of platforms (Jordan 2017; Stuart et al. 2019). It has become common for departments to have an online presence, used to promote research and teaching taking place within them. They are used by professional services as a means of internal communication, directed at staff and students alike. They are even prominent with external actors such as publishers and funders, for whom these platforms provide a means for ongoing interaction with their stakeholders.

The most robust data suggests 30% of academic staff at Russell Group universities are using social media to share updates about their research (Zhu and Purdam 2017). But even this is likely to be an underestimate of present use given increasing uptake in wider society since this research was conducted, as well as the ambiguous character of professional use when one of the defining characteristics of social media platforms is their tendency to blur the boundary between the personal and the professional in ways which are difficult to capture through survey instruments (Boyd 2014; Jordan 2020a). It is significant we now know increasing amounts about how individual academics relate to social media: how many use which platforms, what uses they want to make of them and what problems they perceive in using them. These questions have usually been addressed through survey instruments, e.g. Research Information Network (2010), Rowlands et al. (2011), Lupton (2014) and Van Noorden (2014). There are certainly exceptions to this trend. For example, Shephard et al. (2019) combined a participatory workshop method with 25 interviews, suggesting the range of qualitative methods which can be seen in a number of other papers. However, even these have tended to remain within the framework established by survey research, concerning themselves with *individuals*, their changing practice and their evaluation of these changes. There are methodological reasons for this: mapping the diffusion of and initial reactions to a still novel technology within a professional field is a logical first step for empirical study. The survey is the natural instrument for this and is well established within education technology and information systems research. This inevitably invites qualitative research to flesh out and/or challenge these findings, while remaining within this individualised framework.

This observation should not be read as an attack upon a literature that has identified how social media is used within higher education, as well as the expectations and concerns of users and non-users alike. However, there is a risk research that remains within an *individualised* mode of analysis that makes it difficult to address institutional issues generated by social media. This risks being compounded by the rapidly proliferating grey literature on social media for academics: for every research paper that has been published on the topic, there are countless more blog posts, slide decks and videocasts (etc.) in circulation which exercise a still uncertain influence over social media practice. Its content tends to be both individual and instrumental, concerned with how individual staff might use social media to their professional advantage.

van Dijck and Poell (2018) usefully elucidate the assumptions these two strands of literature share, with their conception of the *social-media-as-tools* approach. These platforms are framed as things which can be picked up and used, with definable consequences for routine activities. So, it becomes a matter of whether social media (either in general or a particular platform) helps or hinders teaching, research, networking etc. This reflects the wider literature on social media and non-tertiary education (Selwyn and Sterling 2016). This is compounded by the aforementioned tendency towards an individualised mode of analysis, narrowing the focus to shifts in individual practice which can register empirically through survey instruments. The *individualised* mode of analysis and *instrumentalised* conception of technology are entangled in the history of social scientific thought, with Gane (2004: 3) identifying the early sociologist Max Weber as the origin of this tendency to reduce technology to the uses which actors make of it and the meanings which this use holds for them (Latour 2005). This obscures the *platform* character of social media while framing it as something *new* intruding from outside, with practice left as a dependent variable that changes for better or worse in response to these tools. This in turn obscures the political economy of social platforms by narrowly analysing tools and practice without consideration of how their uptake and use generates wealth for commercial actors as part of a wider process of capital accumulation (Zuboff 2019).

In the following section, we present a case study of social media in the 2014 Research Excellence Framework (REF) to explore the incorporation of social media into the evaluative infrastructure of higher education, as well as what this means for how universities relate to these platforms in the future. We suggest a neo-institutionalist approach can shed light on these emerging issues.

## Case Study: Social Media and the Impact Agenda

The quality of research in the UK Higher Education sector is periodically assessed through a national auditing of universities’ research outputs. Since 1986, such exercises have been influential in allocation of funding (Jump 2013). Until 2008, this was undertaken on an approximately 5-year cycle via a process known as the Research Assessment Exercise (RAE) (ibid.). The RAE was subject to criticism and was replaced by the Research Excellence Framework (REF), which took place for the first time in 2014. A key distinction between the RAE (which arguably relied too heavily on peer review) and the REF was the foregrounding of the perceived ‘real world’ impact of research, framed as a reflection of better ‘value’ for research funding (ibid.). Whilst such impacts may be challenging to define, *some* academics do perceive its inclusion to be valuable in presenting a richer account of scholarly activity (Watermeyer 2012). It nonetheless remains a contentious exercise which has been widely criticised for its stated mission to reorientate scholarly research, as well as the perceived nebulousness of how ‘impact’ is conceived in practice (Sayer 2014). It should be stressed that the ‘third mission’ of the university (i.e. beyond teaching and research) predates the impact agenda to at least some extent, which suggests we ought to conceive of the latter in terms of *how* academics are encouraged to engage with society rather than the fact of engagement as such (Bacevic 2017). This is why understanding how social media is treated within the REF 2014 is so important because it helps us understand how academic orientations towards this relatively new field of activity are likely to be shaped by the incentives of research assessment. In this sense, we take a critical approach towards the impact agenda, even if we do not problematise it for the purposes of the case study.

The starting point for this inquiry was a question of whether evidence from social media platforms has been called upon when authoring impact case studies, given the slipperiness of the term ‘impact’, and the perceived mediating role of the platforms between content providers and publics (however defined). Following the shift in focus from scholarly outputs to social impact, the 2014 REF was the first assessment exercise to include submissions of ‘impact case studies’. Case studies were intended to make explicit the links between research undertaken and specific impacts, structured according to the following sections: summary of the impact; underpinning research, references to the research, details of the impact and sources to corroborate the impact (Hill 2016). A total of 6975 case studies were submitted as part of the 2014 REF, 6679 of which were subsequently published online in a database (REF 2014). Although the database does not include the results of the assessment for each case study, it nonetheless represents a valuable resource for further research and analysis.

Published analyses of the database fall into two groups: analyses from the perspective of a particular subject and exploration of broader themes related to impact across the data as a whole. Subject-specific approaches have been applied from the perspectives of Anthropology (Simpson 2015), Business (Syed and Davies 2016), Educational Research (Cain and Allan 2017; Kneale et al. 2016); Educational Technology (Jordan 2020b), Engineering (Biri et al. 2014; Robbins et al. 2016), Health (Greenhalgh and Fahy 2015; Hinrichs and Grant 2015; Kamenetzky et al. 2016; Kelly et al. 2016); Leadership, Governance and Management (Ross and Morrow 2016); Library and Information Science (Marcella et al. 2016) and Social Work (Smith and Stewart 2017).

The second category includes studies which interrogate the entire database, to examine trends in relation to the impact agenda more broadly. Examples here include questions around relationships between impact and bibliographic metrics, financial value and public engagement, and what ‘counts’ as impact. It is debatable whether the case studies approach accurately captures research impact (Khazragui and Hudson 2015); as 2014 was the inaugural REF, such cross-cutting studies may help inform how the process is developed in the future. With the addition of data about the scoring received by case studies in the REF, Woolridge (2017) demonstrates a link between highly rated case studies and altmetrics scores, while Ravenscroft et al. argue that the link between impact case studies and traditional citation-based metrics is weak (Ravenscroft et al. 2017). Reed and Kerridge (2017) examine the link between units of assessment which scored entirely 3* or 4* (the highest grades conferred by the REF) for their impact case studies and their funding received, concluding that on average, a 4* case study was worth approximately £35,000 more than a 3* one in funding terms. Loach et al. (2016) surveyed the types of evidence cited within impact case studies, with testimonials, reports and articles being the most frequently used, and some disciplinary preferences emerged.

For these reasons, we expect a sustained correlation between altmetrics scores and highly rate case studies will lead to an organisational prioritisation of the former, even if there is a lack of conclusive evidence about a causal relationship with the latter. The same point can be made about social media as emerging dissemination mechanisms, even if there is widespread recognition that dissemination is insufficient for impact even while it remains necessary. Neo-institutionalism suggests organisations will tend to mimic each other under conditions of uncertainty when working with ambiguous goals (DiMaggio and Powell 1983), e.g. how do we identify ‘research impact’ and how will our attempts to demonstrate it be evaluated by a still unfamiliar apparatus? The emergence of clear ‘winners’ and ‘losers’ from REF 2014 presents criteria for universities to replicate the perceived behaviour of those deemed successful. Furthermore, there is a tendency for organisations operating within the same competitive environment to come to resemble each other by virtue of responding to comparable pressures, as Caplan and boyd (2018) insightfully analyse in the case of print media adapting to the influence of Facebook.

In this sense, a neo-institutionalist analysis would suggest two mechanisms driving what DiMaggio and Powell (1983) term institutional isomorphism in how universities respond to the new criteria of impact: one relating directly to ‘impact’ and the uncertainty which surrounds it and the other relating to the competitive allocation of funding through research evaluation. In light of this, we suggest it is helpful to consider the role social media played in this round of case studies with a view to understanding how it is likely to be seen by universities, directly as a consequence of its clear linkage with altmetrics (with the significant role they accord to attention through social media platforms) and indirectly given the obvious, if hard to pin down, link between academics communicating with extra-academic audiences and their research impact.

The impact of research is often cited as a reason for academics to engage with social media, either through increasing the spread and readership of formal academic publications (Thelwall 2017), or as a mechanism to improve impact through enhanced public engagement (LSE Impact blog 2015). A question relating to social media was presented to the workshops facilitated by the research team at Kings, although it was not deemed to be a high enough priority to warrant inclusion in the study (‘How is social media being used to communicate research and contribute to impact?’; King’s College London and Digital Science 2015: 83). Social media is also mentioned as part of the vast range of types of evidence found in the case studies in the analysis by Loach et al. (2016), although it was not explicitly addressed in the analysis. The neo-institutionalist reasoning above us give us grounds to expect this relative neglect would be reversed in subsequent years and even a cursory inspection of the landscape of doctoral training and research support reveals the ubiquity of references to social media, even if we cannot offer a quantification in this paper.

## Data Collection and Analysis

A two-step approach to data collection and analysis was used. First, a comprehensive sample of case studies which mention social media or specific social media platforms was constructed. Search queries were run on the impact case studies database for 42 terms, of which 13 yielded no results (Table 1). The results of the queries were exported as spreadsheets, tagged with the keywords which featured, combined and duplicates removed. A total of 1675 case studies were included in the resulting sample, which is 25% of the total number of non-redacted REF 2014 case studies (6679 in total). The first and second research questions were addressed by visualising trends in the dataset, and descriptive statistics.

**Table 1:** Search terms used to query the impact case studies database. Note that responses for the terms ‘slack’ and ‘vine’ were false positives

*Search term*	*No. of case studies*	*Search term*	*No. of case studies*
**‘social media’**	**278**	podcast	214
‘academia.edu’	13	reddit	3
bebo	1	researchgate	2
biomedexperts	0	‘second life’	9
blog	678	skype	23
‘del.icio.us’	0	slack	0**
diigo	0	slideshare	2
facebook	227	snapchat	0
figshare	0	soundcloud	7
flickr	13	tinder	0
foursquare	2	tumblr	4
‘google hangout’	0	twitter	233
‘google scholar’	352	vimeo	23
instagram	1	vine	0*
linkedin	14	webinar	26
mendeley	1	whatsapp	1
‘microsoft academic’	4	wikipedia	61
myspace	4	wordpress	32
orcid	0	‘yik yak’	0
periscope	0	youtube	348
pinterest	2	zotero	0

**Fig. 1:**
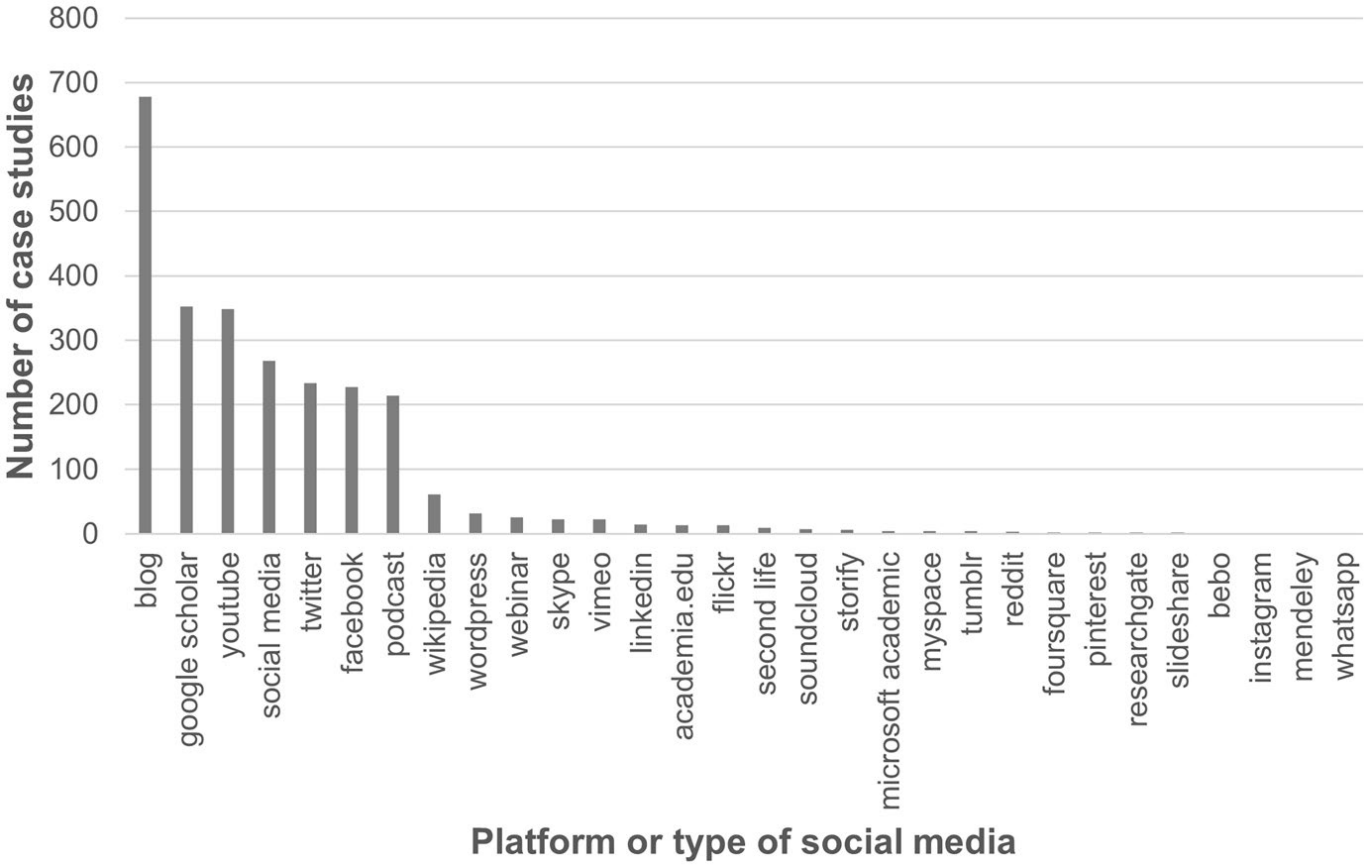
Bar chart showing the frequencies of case studies returned from the REF database in response to a range of social media platform search terms, arranged in descending order of frequency

The first research question asked ‘*Which social media platforms are mentioned in the context of what institutions consider to be exemplary impact research?*’, which was addressed simply through the frequency of case studies returned for each search query. The information presented in Table 1 is also shown graphically in Fig. 1. When presented as a bar chart, two notable characteristics of the sample become clear. First, a wide range of platforms are mentioned at least once within the sample. Second, the frequency of terms is steeply unequal; we see a cluster of few core, high-frequency platforms, followed by a ‘long tail’ of low-frequency platforms (Fig. 1). The high-frequency cluster, all being mentioned in at least 200 case studies, include blogs, Google scholar, YouTube, social media (generalized), Twitter, Facebook and podcasts.

**Fig. 2:**
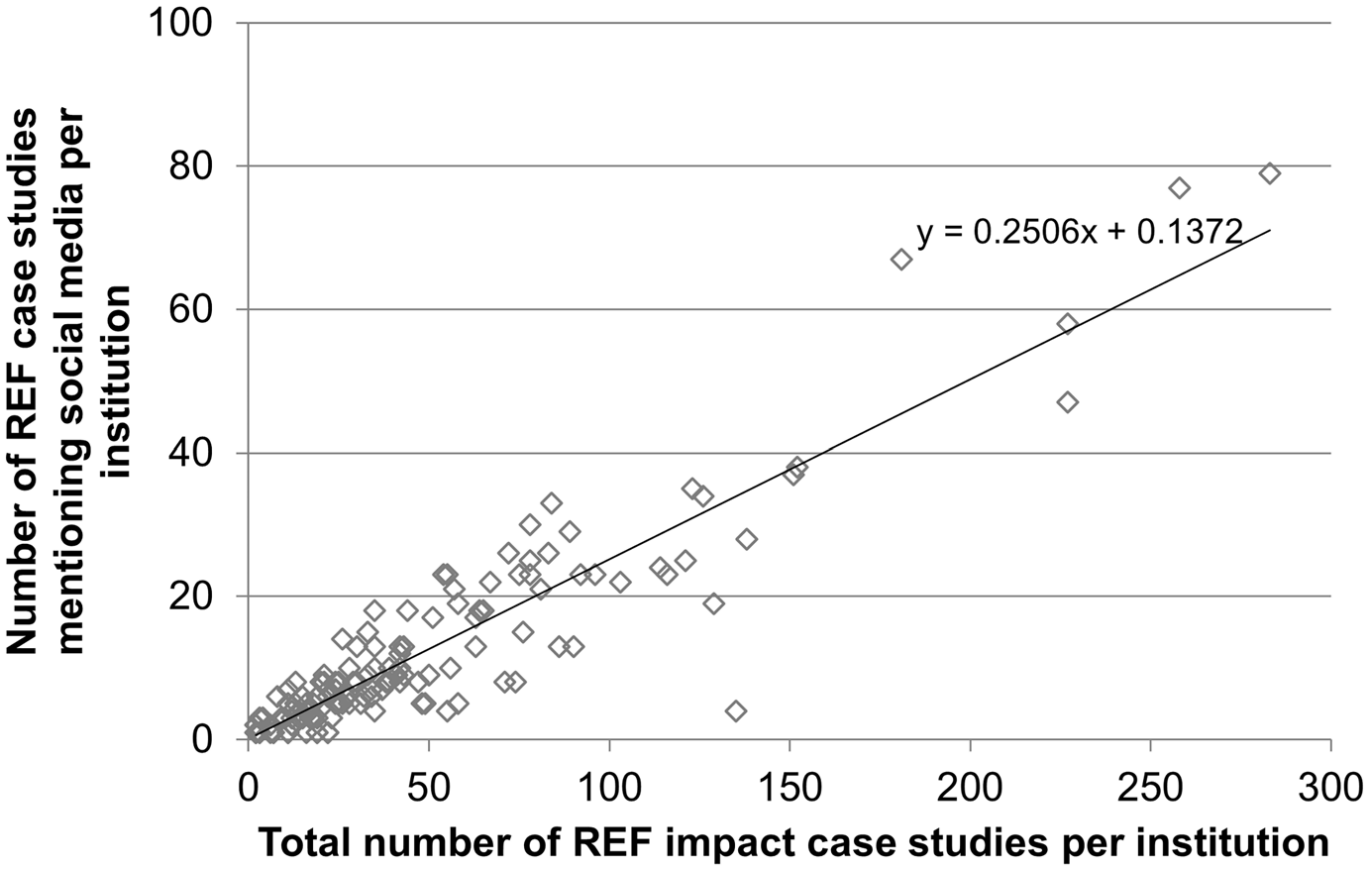
Number of REF case studies included in the sample, plotted against the total number of case studies in the database, per institution

The second question, ‘*How does the sample of impact case studies which mention social media compare to the broader trends in the database?*’, was addressed by examining the dataset in terms of differences in institutions and subject areas. Plotting the total number per institution against number which mentions social media (Fig. 2) shows that there is a fairly consistent pattern overall of around 25% of case studies mentioning social media. It is important to remember that this reflects the diffusion of social media in the period *prior* to the 2014 case studies, when these platforms were less mainstream. The two institutions which deviate to the greatest extent are Imperial College London (social media is notably under-represented; 4 of 135 case studies, or 3%) and the University of Manchester (social media is represented to a greater extent; 67 of 181 case studies, or 37%).

To examine the sample in terms of subject areas, the case studies are categorised according to 36 specific subject topics (‘units of assessment’), which were grouped into four broader disciplinary areas for the REF (panels A through D; the categories are prescribed by the database, http://impact.ref.ac.uk/CaseStudies/). The overall number of case studies per panel and the number which mentioned social media per panel are shown in Table 2. Social media is mentioned much more frequently in Panel D (Arts and Humanities).

**Table 2:** Number of REF impact case studies per panel in the online database in total (‘*N* overall’) and the number mentioning social media (‘*N* sample’)

**Panel**	***N* overall**	***N* sample**	**Percent**
A (~ Biological and Medical Sciences)	1586	207	13.1
B (~ Physical and Mathematical Sciences)	1469	259	17.6
C (~ Social Sciences)	1965	460	23.4
D (~ Arts and Humanities)	1617	749	46.3

In addition to examining the prevalence of different social media platforms and the types of REF impact case studies they are associated with, a sample of 100 case studies drawn at random from those mentioning social media were explored in terms of how social media was being used in this context. Several recurrent themes emerge from our initial analysis, including:

• Tracking of traditional scholarly publishing: citation counts and rankings through sites such as Google Scholar and Microsoft Academic. These are being incorporated into the evaluative infrastructures of higher education.
• Mainstream media reflected through social media: for example, television coverage secondarily made available through YouTube, or academic work being featured in a newspaper’s blog. Social media is being used to expand upon and archive coverage in broadcast and print media.
• Other social media channels: a wide range of third-party organisations (not led by the academics involved in the case studies themselves) which may have featured or referred to the research underpinning the case study. Examples include institutional, political and corporate social media, and Wikipedia pages.
• Academic-led dissemination strategies: encompasses a wide range of social media engagement led by the academics themselves, either as personal or project-based accounts. The main examples include blogs, Twitter accounts and YouTube channels.
• Social media used as a way of involving participants in research: instances found in the subsample involved using social media to directly communicate with participants, such as holding online discussions and soliciting feedback through social media, and also in co-production of research outputs including blog posts and YouTube videos.
• Social media as an application of research: a small but distinct theme, where social media platforms were cited as benefitting from the research reported in the case study. For example, YouTube videos having been made with technology developed in the case study.
• Quantifying impact: figures were often associated with social media mentions in the case studies. Metrics were wide-ranging, and several different metrics could be associated with a single platform in different cases; examples included numbers of comments, followers, views, downloads, visits, participants, likes and mentions.

While a sense of approaching saturation was achieved with the coding, these themes may not be exhaustive as they are derived from a sub-sample of cases. Within the 100 cases analysed, the relative prevalence of the themes varies considerably. For example, relatively few mention social media to involve participants in research, while a surprisingly high proportion simply use social media mentions and metrics as a reflection of traditional scholars’ impact or media appearances.

## Platforms and Institutions in the Post-Pandemic University

If we retain the *individualistic* and *instrumentalist* assumptions we identified earlier in the paper, these findings remain a matter of how individual practice figures into the evaluation of research impact. However, if we approach them through the conceptual framework of the platform offered earlier in the paper, we can identify a number of institutional issues emerging from the characteristics of the platforms:

1. **Data-generating**: the data generated by social media platforms is being incorporated into the evaluative infrastructures of higher education through its inclusion in impact case studies, as well as the allocation of resources which follows from their evaluation. Obviously, it is one of many forms of evidence entering into this process, but it is unusual in that this data is integral to the platform’s business model in three senses: it is treated as an asset by the firm, it is used to operate their advertising business and it is used to regulate the platform in order to maximise user engagement (van Dijck 2013; Zuboff 2019). Furthermore, recent scandals about the reliability of Facebook’s video metrics illustrate how the reliability of this data cannot be taken for granted. The use of this data in impact evaluation *loosely couples* higher education institutions with the data infrastructures of platform capitalism, with uncertain longer-term consequences. This is particularly pronounced with platforms such as Academia.edu, ResearchGate, Google Scholar and Microsoft Academic that purposively take the academy as a site of data generation (Komljenovic 2018).
2. **Opacity**: it is significant the data being incorporated into evaluative infrastructure is *user-facing* rather than *back-end*. As discussed earlier, platforms are Janus-faced infrastructures which combine a user-facing front end system (intended to shape user behaviour, with data such as ‘follower counts’ and ‘retweets’ presented to users as one means through which to do this) with a back-end system (operating with a much wider data set, used to algorithmically modulate what the user is presented with). The reliance on front end metrics in research evaluation (comments, followers, views, downloads etc.) renders back-end operations opaque, conveying the impression of the platform as a neutral mediator of the activity directly recorded in the metrics, rather than an evaluative infrastructure intervening in and shaping the activity taking place. Following Pickering (2010), we could say the metrics ‘ontologically veil’ the platform, preventing it from being represented in a way that could feed into practice or analysis.
3. **Lock in**: The use of these metrics naturalises the categories of the platform, entrenching the widespread sense that ‘followers’, ‘views’, ‘retweets’ and the like are transparent terms with which to talk about online activity. This leaves us with an impoverished view of what Beer (2012) calls the *politics of circulation*, making it harder to think in more sophisticated ways about the network structures and dissemination patterns of social media, as well as the interests being served by these (Margetts 2017). Even if the naive reproduction of metrics is recognised as bad practice, in the sense of *engagement* being insufficient for *impact*, the reproduction of these categories in order to make sense of the communicative affordances of social media reproduces the perspective of social media users in a setting external to social media. In this sense, we begin to think of dissemination in terms of social media, with its imaginary of amplification, virality and networks (Beer 2018).

We should not expect these developments to unfold uniformly across higher education. As can be seen from the discrepancy between units of assessment, social media serves a different purpose in different disciplinary areas. To what extent are disciplines with a weaker position within the academy liable to draw on the perceived affordances of social media in order to demonstrate and develop their impact on wider society? To what extent is social media engagement coming to be seen as a proxy for impact *capacity* by academics and researcher managers? How much diversity is there in how social media are understood by those with a stake in using them for research impact? The neo-institutionalist analysis we have outlined provides strong grounds for expecting growing *isomorphism* over the coming years, as universities respond to the same incentive structure encountered through research assessment while replicating what is perceived as ‘best practice’ given the remaining ambiguity about what impact is and how it can be demonstrated in a way that is evaluated highly. There is much more to this process than social media, but we suggest this is an institutional mechanism through which social media is coming to be incorporated into higher education in a way that transcends the level of individual practice and its relationship to a novel technology.

There are obvious limitations to our capacity to make claims in the present tense based on data which was produced in the previous decade. In part, this is an artefact of research assessment cycles which mean we inevitably work with data produced from the last cycle, even if we are nearing the end of the cycle which came after it. This problem is compounded by the time it takes for the data to be released for public analysis. However, it nonetheless raises the question of how social platforms have changed during this time, as well as what this means for their use within higher education. It is beyond the scope of our paper to offer a systematic account of this, but there are three factors important to consider. Firstly, the evidence base suggests that use of social media by academics has grown since the end of the last research assessment cycle, even if it remains curiously difficult to produce a precise estimate given the fuzzy boundaries of what counts as ‘social media’ and what it means for an academic to ‘use’ social media in a professional capacity (Carrigan 2019a, b). This obviously reflects the sustained growth of social media within and across populations, from 2.86 billion in 2017 through to 3.78 billion in 2021, but it also tracks a sustained normalisation of social media within higher education (Statista 2021). This is not a uniform trend across nations and regions: we still lack an empirical literature which analyses this variation, but the heavily interconnected nature of research communities (as a consequence of scholarly associations, collaboration networks and publications with international readership) means that we should not overstate the significance of this variation. Secondly, the Covid-19 pandemic has intensified reliance upon digital platforms across social life as a means to facilitate social distancing, including within higher education (Carrigan and Fatsis 2021). It should be stressed that this reliance highlighted social inequalities concerning who could work from home and who could not, but the fact this was not a uniform trend does not detract from its organisational significance. This enforced reliance upon digital platforms can be expected to accelerate the existing trend towards platformisation within the academy, even if the use of social media by academic staff for research communication was relatively peripheral in comparison to what was widely termed the ‘online pivot’. Even if the end of the pandemic, which is not imminent at the time of writing, brings some roll back of the emergency measures and everyday practices adopted during the crisis, it seems implausible to expect a complete return to the ‘old normal’ due to the accretion of changes over a number of years. For these reasons, we suggest our analysis has become *more* relevant with the time since the data was collected because social platforms have come to play a more prominent role in the intervening years (Robertson 2018). It offers a way to think about these salient issues in a manner which avoids the individualistic bias within the existing literature, foregrounding the centrality of institutions in how social media brings about change within universities.

## Conclusion

In the time we have been working on this paper, the Covid-19 pandemic has transformed the world in ways which will have consequences for decades to come. The argument we had sought to make was that we urgently need to understand the role of platforms within higher education because this is only likely to grow with time. Our claim is that if we restrict our focus to individual practice and how individuals use technology then we would miss a broader institutional transformation: how *platformisation*, the insert of platforms as intermediaries into a process, changes the character of the mediated activity and exercises an influence over the organisations in which that mediation takes place. The fact we now rely on platforms for the bulk of activity within higher education only renders it more urgent that we understand this transition. The university, as an institution, is now dependent on platforms to the extent it seeks to operate at a distance. There are theoretical, methodological and empirical challenges posed by this which we have only begun to scratch the surface of in this paper.

The case study we have presented of social media in the 2014 REF impact case studies explores a particular category of platforms in a particular area of university life. We hope it also illustrates how to think of platforms in higher education in institutional terms rather than as simply a matter of technology. The full contours of this crisis remain uncertain as we write this midway through 2020, but it seems clear we will confront a radically changed world at the end of it (Fuchs 2020). This leaves us with another question: *what will the post-pandemic university be like*? It seems likely it will be a ‘platform university’ in which our dependence on these infrastructures is ubiquitous, normalised and planned for (Carrigan 2019a; Robertson 2018). If this prediction proves to be correct, then *platforms* and *institutions* will need to be key categories in higher educational research in the coming years.

## Footnotes

1 Our thanks to the anonymous reviewer for suggesting this important point.

2 There is a parallel but distinct story to be told in other university systems.

## References

[CR1] Andrejevic, M. (2013). *Infoglut: How too much information is changing the way we think and know.* London: Routledge. 10.4324/9780203075319.

[CR2] Bacevic, J. (2017). Beyond the third mission: towards an actor-based account of universities’ relationship with Society. In H. Ergul & S. Cosar (Eds.), *Universities in the Neoliberal Era* (pp. 21–39). London: Palgrave. 10.1057/978-1-137-55212-9.

[CR3] Bacevic, J. (2019). With or without U? Assemblage theory and (de) territorialising the university. *Globalisation, Societies and Education, 17*(1), 78-91. 10.1080/14767724.2018.1498323.

[CR4] Beer, D. (2012). Open Access and academic publishing: some lessons from music culture. *Political Geography, 31*(8), 479-480. 10.1016/j.polgeo.2012.08.001.

[CR5] Beer, D. (2018). *The Data Gaze: Capitalism, power and perception*. London: Sage. 10.4135/9781526463210.

[CR6] Biri, D., Oliver, K., & Cooper, A. (2014). What is the impact of BEAMS research? An evaluation of REF impact case studies from UCL BEAMS. London: UCL. http://discovery.ucl.ac.uk/1458641/1/Biri_impact-report-dec14.pdf. Accessed 26 July 2021.

[CR7] Boyd, D. (2014). *It's complicated: The social lives of networked teens*. New Haven, CO: Yale University Press.

[CR8] Cain, T., & Allan, D. (2017). The invisible impact of educational research. *Oxford Review of Education, 43*(6), 718-732. 10.1080/03054985.2017.1316252.

[CR9] Caplan, R., & boyd, d. (2018). Isomorphism through algorithms: Institutional dependencies in the case of Facebook. *Big Data & Society, (5)*1, 1-12. 10.1177/2053951718757253.

[CR10] Carrigan, M. (2018). The evisceration of the human under digital capitalism. In I. Al-Amoudi & J. Morgan (Eds.), *Realist Responses to Post-Human Society: Ex Machina* (pp. 175-191). London: Routledge.

[CR11] Carrigan, M. (2019a). The Platform University. Discover Society*,* (68). https://archive.discoversociety.org/2019/05/01/focus-the-platform-university/. Accessed 26 July 2021.

[CR12] Carrigan, M. (2019b). *Social Media for Academics. 2*^*nd*^Ed. London: Sage.

[CR13] Carrigan, M., & Fatsis, L. (2021). *The Public and their Platforms*. Bristol: University of Bristol Press.

[CR14] DiMaggio, P. J., & Powell, W. W. (1983). The iron cage revisited: Institutional isomorphism and collective rationality in organizational fields. *American Sociological Review*, *48*(2), 147-160. 10.2307/2095101.

[CR15] Fuchs, C. (2020). Everyday life and everyday communication in coronavirus capitalism. *tripleC: Communication, Capitalism & Critique. Open Access Journal for a Global Sustainable Information Society, 18*(1), 375–399. 10.31269/triplec.v18i1.1167.

[CR16] Gane, N. (2004). *The Future of Social Theory*. London: Continuum.

[CR17] Gillespie, T. (2010). The politics of ‘platforms’. *New Media & Society, 12*(3), 347-364. 10.1177/1461444809342738.

[CR18] Gillespie, T. (2018). *Custodians of the Internet: Platforms, content moderation, and the hidden decisions that shape social media*. New Haven, CO: Yale University Press. 10.12987/9780300235029.

[CR19] Gitelman, L. (2013). *Raw data is an oxymoron*. Cambridge, MA: MIT press. 10.7551/mitpress/9302.001.0001.

[CR20] Goldenfein, J., Benthall, S., Griffin, D. S., & Toch, E. (2019). Private Companies and Scholarly Infrastructure–Google Scholar and Academic Autonomy. Digital Life Initiative. https://www.dli.tech.cornell.edu/post/private-companies-and-scholarly-infrastructuregoogle-scholar-and-academic-autonomy. Accessed 21 October 2021.

[CR21] Greenhalgh, T., & Fahy. N. (2015). Research impact in the community-based health sciences: an analysis of 162 case studies from the 2014 UK Research Excellence Framework. *BMC Medicine, 13*, 232. 10.1186/s12916-015-0467-4. 10.1186/s12916-015-0467-4 PMC4578424 26391108

[CR22] Hinrichs, S., & Grant, J. (2015). A new resource for identifying and assessing the impacts of research. *BMC Medicine*, *13*, 148. 10.1186/s12916-015-0364-x. 10.1186/s12916-015-0364-x PMC4479084 26108576

[CR23] Hill, S. (2016). Assessing (for) impact: future assessment of the societal impact of research. *Palgrave Communications, 2*, 16073. 10.1057/palcomms.2016.73.

[CR24] Jordan, K. (2017). Examining the UK Higher Education sector through the network of institutional accounts on Twitter. *First Monday, 22*(5), 5. 10.5210/fm.v22i5.7133.

[CR25] Jordan, K. (2019). From social networks to publishing platforms: a review of the history and scholarship of academic social network sites. *Frontiers in Digital Humanities*. 10.3389/fdigh.2019.00005.

[CR26] Jordan, K. (2020a). Imagined audiences, acceptable identity fragments and merging the personal and professional: how academic online identity is expressed through different social media platforms. *Learning, Media and Technology*, *45*(2), 165-178. 10.1080/17439884.2020.1707222.

[CR27] Jordan, K. (2020b). Educational technology and research impact: the two roles of e-learning and related terms in the 2014 REF impact case studies. *Research in Learning Technology*, *28*. 10.25304/rlt.v28.2306.

[CR28] Jump, P. (2013). Evolution of the REF. Times Higher Education, 17 October. https://www.timeshighereducation.com/features/evolution-of-the-ref/2008100.article. Accessed 26 July 2021.

[CR29] Kamenetzky, A., Hinrichs-Krapels, S., Wooding, S., & Grant, J. (2016). An analysis of the impact of research supported by the UK National Institute of Health Research. In A. Kamenetzky, S. Hinrichs-Krapels, S. Wooding, & J. Grant (Eds.), *Impacts of agricultural research - an approach of societal values: An International Conference organized by the French National Institute for Agricultural Research (INRA)*. London: The Policy Institute at King's. https://kclpure.kcl.ac.uk/portal/files/52658433/Impact_of_NIHR_research.pdf. Accessed 26 July 2021.

[CR30] Kelly, D., Kent, B., McMahon, A., Taylor, J., & Traynor, M. (2016). Impact case studies submitted to REF 2014: The hidden impact of nursing research. *Journal of Research in Nursing*, *21*(4), 256-268. 10.1177/1744987116646153.

[CR31] Khazragui, H., & Hudson, J. (2015). Measuring the benefits of university research: impact and the REF in the UK. *Research Evaluation, 24*(1), 51-62. 10.1093/reseval/rvu028.

[CR32] King’s College London and Digital Science. (2015). The nature, scale and beneficiaries of research impact: An initial analysis of Research Excellence Framework (REF) 2014 impact case studies*.* Bristol, United Kingdom: HEFCE. http://www.hefce.ac.uk/media/HEFCE,2014/Content/Pubs/Independentresearch/2015/Analysis,of,REF,impact/Analysis_of_REF_impact.pdf. Accessed 26 July 2021.

[CR33] Kitchin, R. (2014). *The data revolution: Big data, open data, data infrastructures and their consequences*. London: Sage. 10.1111/jors.12293.

[CR34] Kneale, P., Cotton, D. R. E., & Miller, W. (2016). REF 2014: Higher education pedagogic research and impact. http://www.wlv.ac.uk/media/departments/directorate-of-academic-support/documents/colt/pedrg/REF-2014-higher-education-pedagogic-research-and-impact-(1)-(3).pdf. Accessed 26 July 2021.

[CR35] Komljenovic, J. (2018). Big data and new social relations in higher education: Academia.edu, Google Scholar and ResearchGate. In R. Gorur, S. Sellar & G. Steiner-Khamsi (Eds.), *World Yearbook of Education 2019: Comparative Methodology in the era of big Data and Global Networks* (pp.169–186). London: Routledge. 10.4324/9781315147338-14.

[CR36] Latour, B. (2005). *Reassembling the social: An introduction to actor-network theory.* Oxford: Oxford University Press. 10.1080/10967490701515606.

[CR37] Loach, T., Adams, J., & Szomszor, M. (2016). Digital research report: The societal and economic impacts of academic research – International perspectives on good practice and managing evidence. *Digital Science*. 10.6084/m9.figshare.3117928.v2.

[CR38] LSE Impact blog. (2015). Reading list: Using social media for research collaboration and public engagement. http://blogs.lse.ac.uk/impactofsocialsciences/2015/06/26/reading-list-using-social-media-for-research/. Accessed 26 July 2021.

[CR39] Lupton, D. (2014). *Digital sociology*. London: Routledge. 10.4324/9781003116974-25.

[CR40] Marcella, R., Lockerbie, H., & Bloice, L. (2016). Beyond REF 2014: The impact of impact assessment on the future of information research. *Journal of Information Science*, *42*(3), 369-385. 10.1177/0165551516636291.

[CR41] Margetts, H. (2017). Political behaviour and the acoustics of social media. *Nature Human Behaviour, 1*(4), 1-3. 10.1038/s41562-017-0086.

[CR42] Marres, N. (2017). *Digital sociology: The reinvention of social research*. Cambridge: Polity Press.

[CR43] Marres, N. (2018). Why we can't have our facts back. *Engaging Science, Technology, and Society, 4*, 423–443. 10.17351/ests2018.188.

[CR44] McAfee, A., & Brynjolfsson, E. (2017). *Machine, platform, crowd: Harnessing our digital future*. New York: WW Norton & Company.

[CR45] McCluskey, F. B., & Winter, L. M. (2012). *The idea of the digital university: Ancient traditions, disruptive technologies and the battle for the soul of higher education*. Washington, DC: Westphalia Press.

[CR46] Mayer-Schönberger, V., & Cukier, C. (2013). *Big data: A revolution that will transform how we live, work, and think*. Boston, MA: Houghton Mifflin Harcourt.

[CR47] Nash, K. (2019). Neo-liberalisation, universities and the values of bureaucracy. *The Sociological Review, 67*(1), 178-193. 10.1177/0038026118754780.

[CR48] Pasquale, F. (2016). Two narratives of platform capitalism. *Yale Law & Policy Review, 35*(1), 309. https://ylpr.yale.edu/two-narratives-platform-capitalism. Accessed 20 October 2021.

[CR49] Pickering, A. (2010). *The cybernetic brain: Sketches of another future*. Chicago, IL: University of Chicago Press. 10.7208/chicago/9780226667928.001.0001.

[CR50] REF (2014) Impact case studies. http://impact.ref.ac.uk/CaseStudies/. Accessed 26 July 2021.

[CR51] Ravenscroft, J., Liakata, M., Clare, M., & Duma, D. (2017). Measuring scientific impact beyond academia: An assessment of existing impact metrics and proposed improvements. *PLoSONE*, *12*(3), e0173152. 10.1371/journal.pone.0173152. 10.1371/journal.pone.0173152 PMC5344357 28278243

[CR52] Reed, S., & Kerridge, S. (2017). How much was an impact case study worth in the UK Research Excellence Framework? Fast Track Impact blog. http://www.fasttrackimpact.com/single-post/2017/02/01/How-much-was-an-impact-case-study-worth-in-the-UK-Research-Excellence-Framework. Accessed 26 July 2021.

[CR53] Research Information Network (2010). If you built it, will they come? How researchers perceive and use web 2.0. http://wrap.warwick.ac.uk/56246/1/WRAP_Procter_If%20you%20build%20it%20will%20they%20come.pdf. Accessed 21 October 2021.

[CR54] Robbins, P. T., Wield, D., & Wilson, G. (2016). Mapping engineering and development research excellence in the UK: An analysis of REF2014 impact case studies. *Journal of International Development*, *29*(1), 89–105. 10.1002/jid.3255.

[CR55] Robertson, S. L. (2018). Comparing platforms and the new value economy in the academy. In R. Gorur, S. Sellar & G. Steiner-Khamsi (Eds.). *World Yearbook of Education 2019: Comparative Methodology in the era of big Data and Global Networks* (pp. 169–186). London: Routledge. 10.4324/9781315147338-14.

[CR56] Ross, F., & Morrow, E. M. (2016). Mining the REF impact case studies for lessons on leadership, governance and management in Higher Education. LSE Impact blog. http://blogs.lse.ac.uk/impactofsocialsciences/2016/06/08/leadership-governance-and-management-research-mining-the-ref-impact-case-studies/. Accessed 26 July 2021.

[CR57] Rowlands, I., & Nicholas, D., Russel, B., Canty, N., & Watkinson, A. (2011). Social media use in the research workflow. *Learned Publishing, 24*(3), 183-195. 10.1087/20110306.

[CR58] Sayer, D. (2014). *Rank hypocrisies: The Insult of the REF*. London: Sage.

[CR59] Selwyn, N., & Stirling, E. (2016). Social media and education… now the dust has settled. *Learning, media and technology, 41*(1), 1-5. 10.1080/17439884.2015.1115769.

[CR60] Simpson, B. (2015). Ref 2014 and impact: Reading the runes for Anthropology in Action. *Anthropology in Action*, *22*(2), 1-4. 10.3167/aia.2015.220201.

[CR61] Shephard, K., Brown, K., Guiney, T., Dealer, L., & Hesson, G. (2019). Exploring the use of social media by community-engaged university people. *Innovations in Education and Teaching International, 56*(5), 558-568. 10.1080/14703297.2018.1557069.

[CR62] Smith, K. E., & Stewart, E. (2017). We need to talk about impact: Why social policy academics need to engage with the UK's research impact agenda. *Journal of Social Policy*, *46*(1), 109-127. 10.1017/S0047279416000283.

[CR63] Srnicek, N. (2017). *Platform capitalism*. Cambridge: Polity Press.

[CR64] Statista (2021). Number of social network users worldwide from 2017 to 2025. https://www.statista.com/statistics/278414/number-of-worldwide-social-network-users/. Accessed September 6th 2021.

[CR65] Stuart, E., Thelwall, M., & Stuart, D. (2019). Which image types do universities tweet? *First Monday*, *24*(3). 10.5210/fm.v24i3.9225.

[CR66] Syed, J., & Davies, J. (2016). Diversity in the authorship of journal articles and REF impact case studies: How are UK business schools shaping up? In BAM2016 Conference Proceedings.

[CR67] Thelwall, M. (2017). *Web indicators for research evaluation: A practical guide*. San Rafael, CA: Morgan & Claypool. 10.2200/S00733ED1V01Y201609ICR052.

[CR68] van Dijck, J. (2013). *The culture of connectivity: A critical history of social media*. Oxford: Oxford University Press. 10.1093/acprof:oso/9780199970773.001.0001.

[CR69] van Dijck, J., & Poell, T. (2018). Social media platforms and education. In J. Burgess, A. Marwick & T. Poell (Eds.) *The SAGE Handbook of Social Media* (pp. 579–591). London: Sage. 10.4135/9781473984066.

[CR70] Van Noorden, R. (2014). Online collaboration: Scientists and the social network. *Nature news, 512*(7513), 126. 10.1038/512126a. 10.1038/512126a 25119221

[CR71] Watermeyer, R. (2012). Issues in the articulation of ‘impact’: the responses of UK academics to ‘impact’ as a new measure of research assessment. *Studies in Higher Education*, *39*(2), 359-377. 10.1080/03075079.2012.709490.

[CR72] Wellman, B., & Rainie, L. (2012). *Networked*. Cambridge, MA: MIT Press. 10.7551/mitpress/8358.001.0001.

[CR73] Williamson, B. (2018). The hidden architecture of higher education: building a big data infrastructure for the ‘smarter university’. *International Journal of Educational Technology in Higher Education, 15*(1), 1-26. 10.1186/s41239-018-0094-1.

[CR74] Williams, J. (2018). *Stand out of our light: freedom and resistance in the attention economy*. Cambridge: Cambridge University Press. 10.1017/9781108453004.

[CR75] Woodcock, J. (2018). Digital labour in the university: understanding the transformations of academic work in the UK. *tripleC: Communication, Capitalism & Critique. Open Access Journal for a Global Sustainable Information Society, 16*(1), 129–142. 10.31269/triplec.v16i1.880.

[CR76] Woolridge, J. (2017). Altmetrics linked to 3* and 4* impact scores in REF2014 impact case studies. Altmetric blog. https://www.altmetric.com/blog/altmetrics-ref2014-impact-case-studies/. Accessed 26 July 2021.

[CR77] Zhu, Y., & Purdam, K. (2017). Social media, science communication and the academic super user in the United Kingdom. *First Monday, 22*(11). 10.5210/fm.v22i11.7866.

[CR78] Zuboff, S. (2019). *The age of Surveillance Capitalism: The fight for a human future at the new frontier of power.* New York, NY: Profile Books. 10.1080/24701475.2019.1706138.

